# Supplementary material for: Autophagy Promotes Enrichment of Raft Components within Extracellular Vesicles Secreted by Human 2FTGH Cells
Source: Int J Mol Sci. 2024 Jun 4;25(11):6175. doi: 10.3390/ijms25116175 (PMC11172899; doi:10.3390/ijms25116175)
Supplement: Supplementary file 1 [file ijms-25-06175-s001.zip › ijms-2951366-supplementary.pdf]

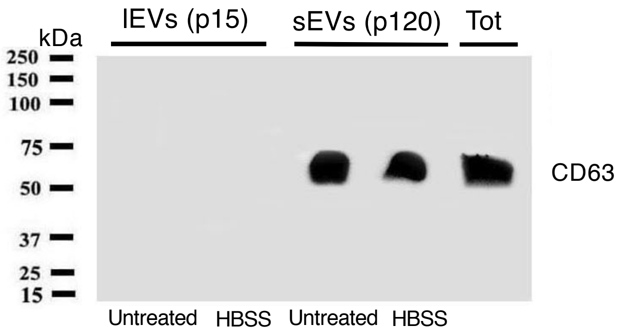

Supplementary Figure S1. CD63 analysis of small and large EVs released by HT1080-derived 2FTGH cells after autophagy stimulation.

Immunoblots of large EVs (lEVs), and small EVs (sEVs) fractions from cells, untreated or treated with HBSS, using anti-CD63 antibodies. The observed MW may vary in the range of 25-65, *(predicted MW: 26 kDa), depending on post translational modifications, post translational cleavages, relative charges and other experimental factors, including unreducing condition. Therefore there really is no consensus molecular weight. A representative experiment among 3 is shown.

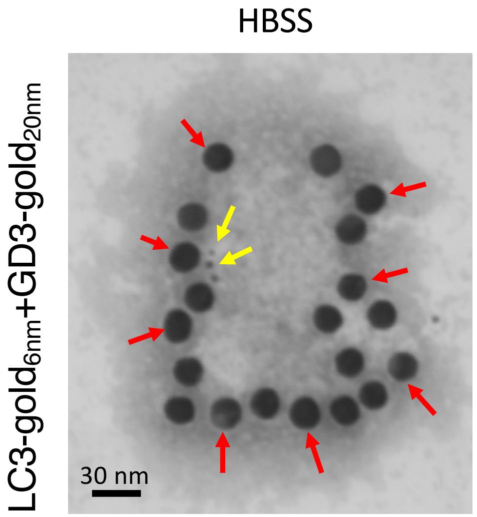

Supplementary Figure S2. Stimulation of autophagy leads to the association of LC3-II and GD3 within small EVs. Representative TEM image of the immunogold double labelling of sEVs from 2FTGH cells treated with HBSS for 4 h at 37°C (scale bar 30 nm) to detect LC3-II (6 nm gold particles, yellow arrows) and GD3 (20 nm gold particles, red arrows) at the EV surface.
